# Plasma lncRNA profiling identified BC200 and NEAT1 lncRNAs as potential blood-based biomarkers for late-onset Alzheimer’s disease

**DOI:** 10.17179/excli2022-4764

**Published:** 2022-05-09

**Authors:** Majid Khodayi, Mohammad Khalaj-Kondori, Mohammad Ali Hoseinpour Feizi, Mortaza Jabarpour Bonyadi, Mahnaz Talebi

**Affiliations:** 1 Department of Animal Biology, Faculty of Natural Sciences, University of Tabriz, Tabriz, Iran; 2 Neurosciences Research Center, Tabriz University of Medical Sciences, Tabriz, Iran

**Keywords:** Alzheimer's disease, memory disorders, gene expression profile, long non-coding RNA, biomarker, early diagnosis

## Abstract

Long non-coding RNAs (lncRNA) play critical roles in pathogenesis of neurodegenerative diseases. Human plasma carries lncRNAs that are stable in the blood, and their disease-specific profile have made them valuable biomarkers for some diseases. This study reports screening of the plasma levels of 90 lncRNAs in patients with Alzheimer disease (AD) to find out plasma-based AD biomarkers. Total RNA was isolated from plasma samples of 50 AD and 50 matched healthy controls. The plasma samples of 10 advanced AD patients and 10 matched healthy controls were screened for expression levels of 90 lncRNAs using Human LncRNA Profiler qPCR Array Kit (SBI). Based on the profiling results, lncRNAs BC200, NDM29, NEAT1, FAS-AS1 and GAS5-AS1 were selected for further analysis in all samples and their biomarker potency was evaluated by ROC curve analysis. We further surveyed RNAseq data by *in silico* analysis. We found that the NEAT1 and BC200 levels in the plasma of the AD patients were significantly higher compared with the control group (P=0.0021, p= 0.02, respectively). ROC curve analysis showed that the plasma level of NEAT1 and BC200 discriminated AD patients from healthy controls with sensitivity of 72 % and 60 %, and specificity of 84 % and 91 % respectively. Moreover, NEAT1 discriminated MCI (60 % sensitivity and 91 % specificity) and advanced-AD patients from healthy controls (73 % sensitivity and 71 % specificity). Besides, plasma level of BC200 discriminated the pre-clinical subjects from healthy controls with 83 % sensitivity and 66 % specificity. A positive correlation was also observed between plasma levels of BC200 with the age patients (r = 0.34, p=0.02). In silico RNAseq data analysis showed that a total of 33 lncRNAs were up-regulated but 13 lncRNAs were down-regulated significantly in AD patients compared with the healthy controls. In conclusion, this study elucidated that the plasma levels of lncRNAs NEAT1 and BC200 might be considered as potential blood-based biomarkers for AD development and progression.

## Introduction

Alzheimer's disease (AD) is the most common form of age-related neurodegenerative diseases manifested by the progressive deterioration of memory and behavioral impairment (Li et al., 2021[20]). A number of cellular and molecular changes such as genetic alterations, are involved in the development of AD (Crews and Masliah, 2010[7]; Guo et al., 2020[14]; Mehdizadeh et al., 2019[27]; Talebi et al., 2020[35]). The pathological hallmarks of AD are the accumulation of amyloid-β (Aβ) plaques and neurofibrillary tangles. According to the 2021 Alzheimer's disease facts and figures, about 6.2 million Americans aged 65 and older suffered from AD or a related dementia disease, and the prevalence of AD was predicted to become tripled by 2050 (Zhang et al., 2021[42]). Unfortunately, currently there is not any therapeutic intervention for stopping AD or delaying its progression, and it is believed that the cost of AD on public health and society will be insufferable (Silva et al., 2019[33]; Yang et al., 2017[40]). Therefore, there is an urgent need for identification of suitable molecular biomarkers that may help in early diagnosis of AD and/or treatment at early prodromal stages of AD. 

Long non-coding RNAs (lncRNAs) are RNA molecules longer than 200 nucleotides, with similarity in structure to mRNAs, but lack protein-coding ability (Luo and Chen, 2016[24]; Zhang et al., 2019[41]). It has been found that lncRNAs play important roles in various biological and cellular processes, including genetic imprinting, embryonic development, cell differentiation, transcriptional and post-transcriptional regulation (Quinn and Chang, 2016[29]; Schmitz et al., 2016[32]). In addition, it is now known that dysregulations and mutations of lncRNAs play a role in a wide range of diseases, from various cancers to neurodegenerative diseases (Wang et al., 2016[39]; Zhang et al., 2020[43]). In addition, due to stability and convenience of detection in plasma, lncRNAs could serve as useful biomarkers that can be adapted for non-invasive (Abdolmaleki et al., 2021[1]) diagnostic approaches for some diseases (Wang et al., 2016[39], 2017[38]). It is increasingly recognized that lncRNAs are tightly related to the development and progression of AD (Faghihi et al., 2008[9]; Garofalo et al., 2021[13]). Expression profiling studies identified hundreds of dysregulated AD-associated lncRNAs in hippocampal region from AD patients in human (Crist et al., 2021[8]; Magistri et al., 2015[25]) and rat models (Tang et al., 2019[36]). Fotuhi and colleagues assessed the BACE1-AS level in plasma samples and showed that it was significantly increased in AD patients compared to the normal individuals (Fotuhi et al., 2019[11]). It has been reported that NEAT1 expression was remarkably up-regulated in Aβ-treated SH-SY5Y and SK-N-SH cells, and its knockdown showed protective effects on the Aβ-induced neuronal damage and attenuated cells' apoptosis (Ke et al., 2019[18]; Zhao et al., 2019[44]). Li et al. reported that BC200 expression was enhanced in the brain of AD patients, suggesting a potential role for BC200 in the AD pathogens (Li et al., 2018[21]). Knockdown of lncRNA BC200 in SH-SY5Y cell model led to an attenuation in BACE1 levels, reduced cell apoptosis, and enhanced cell viability (Li et al., 2018[21]). Some researchers have focused on circulating miRNAs as potent diagnostic biomarker for AD. In APP_Swe_/PS1 mice, serum level of miR-137 was down-regulated compared with normal mice and may serve as a useful noninvasive biomarker for AD (Jiang et al., 2018[17]). In human cells, it was found that miR-298 negatively correlated with both APP and protein level of BACE1 (Chopra et al., 2021[5]). Despite these findings, lncRNA expression profiles in plasma of people with AD are lacking and remain to be explored.

In this study, we first evaluated the expression profile of 90 lncRNAs using the Human LncRNA Profiler qPCR Array Kit (SBI) to identify the lncRNAs with differed plasma levels in 10 patients with advanced Alzheimer's disease and 10 healthy people. In the next step, plasma levels of the candidate lncRNAs were further assessed in all samples, and their potential as biomarkers for AD were investigated. Furthermore, the GSE53697 dataset from the gene expression omnibus (GEO) database was reanalyzed to understand whether it has overlaps with our findings.

## Materials and Methods

### Study subjects and preparation of plasma samples

In this study 50 patients with LOAD (age > 65 years) and 50 healthy voluntaries (age > 65 years) matched for age and sex, were included. The patient recruitment and written informed consent were obtained from all individuals in accordance with the approved guidelines from the Neurology Department at Imam Reza Hospital of Tabriz University of Medical Sciences. All of the subjects were evaluated by a neuroscience specialist, based on the National Institute of Neurological and Communicative Disorders and Stroke and the Alzheimer's Disease and Related Disorders Association criteria (NINCDS-ADRDA) (McKhann et al., 1984[26]). The disease stage was assessed by the Mini-Mental State Examination (MMSE) score to verify any cognitive deficit in both groups. Subjects with a family history of AD, other neurological illnesses including hypothyroidism, alcoholism, hepatitis, spastic lesions, traumatic brain injury, encephalitis, frontal lobe dementia, and Lewy body dementia were excluded from both groups. Peripheral blood (6 ml) was collected from each subject in EDTA-treated tubes and immediately subjected to the three-spin protocol (1500 rpm for 30 min, 3000 rpm for 5 min, and 4500 rpm for 5 min) to collect plasma, fractioned into multiple aliquots, and stored at - 80 °C until further analysis. 

### RNA isolation and lncRNA screening 

Total RNA was extracted from plasma by RiboExTM LS (GeneALL) according to the manufacturer's protocol. Briefly, 250 μl plasma was mixed with 750 μl of the reagent and gently homogenized for 5 min. 2 ml of chloroform was used to separate the homogenate in the aqueous and organic phases. The plasma was precipitated by 500 μl isopropanol and washed with 75 % ethanol. Quality and quantity of the RNA samples were determined using a NanoDrop 2000. The expressions of 90 lncRNAs were analyzed using the commercially available Human LncRNA Profiler qPCR Array Kit (SBI, Mountain View, CA, USA). The qPCR array plate contains assays for 90 lncRNAs and also includes five housekeeping genes (18S rRNA, RNU43, GAPDH, LAMIN A/C, and U6) and one sample failed array detection. Briefly, 5 µL isolated RNA was mixed with reagents (PolyA Buffer, MnCl2, ATP and PolyA Polymerase) to polyadenylate all lncRNAs and incubated at 37 °C for 30 min. Then the oligo dT adaptor and random primers were added and the reactions incubated at 42 °C for 60 min, and heated at 95 °C for 10 min. Finally, the amplification reactions were performed in a Step One Plus real-time PCR system (Applied Biosystems) for 40 cycles (95 °C for 15 sec, 60 °C for 1 min) after an initial 10 min incubation at 95 °C. The relative lncRNA expression was described as fold-change using the 2^−ΔΔCt^ method (Livak and Schmittgen, 2001[22]). 

### Quantitative real-time PCR

Total RNA was reverse transcribed to cDNA using ExcelRT™ cDNA synthesis kit (SMOBIO) with the following conditions: 60 min at 37 °C, and 5 s at 85 °C. The Q-PCR was performed to measure the expression levels of BC200, NDM29, NEAT1, FAS-AS1 and GAS5-AS1 using Step One Plus Real-Time PCR system (Applied Biosystems). RNU6 was used as an internal control to provide normalization. The 2^−ΔΔCt^ method was used to calculate the relative expression. The primer sequences used in this study were listed in Table 1(Tab. 1). 

### ApoE genotyping

Genomic DNA was extracted from peripheral white blood cells by salting out method. Amplification of a fragment encompassing both SNPs rs429358 and rs7412 was done by a specific primer pair (Table 1(Tab. 1)). The PCR products were sequenced and the APOE genotypes of AD patients were identified.

### RNA-seq data analysis

The NGS data used in the present study were downloaded from the NCBI GEO database (https://www.ncbi.nlm.nih.gov/geo/query/acc.cgi). Two datasets, GSE136243 consisted of 164 plasma samples from AD patients, and GSE53697 consisted of human cerebral cortex tissues from nine patients and eight controls (Scheckel et al., 2016[31]), were analyzed. Differentially expressed lncRNA genes were identified using the DeSeq2 R package (Love et al., 2014[23]). The RNA-seq data were aligned into the human reference genome (Hg38 version) by using the Feature Counts package. Genes with log fold changes ≥1 and adjusted p-value ≤0.05 were considered as up-regulated, and the ones with log fold changes ≤0 and adjusted p-value ≤0.05 were considered as down-regulated genes.

### GO functional annotation and KEGG pathway analysis 

Gene ontology (GO) functional annotation and Kyoto Encyclopedia of Genes and Genomes (KEGG) pathway analysis were applied using the Database for Annotation, Visualization and Integrated Discovery (DAVID) tool (Huang et al., 2007[15]) (https://david.ncifcrf.gov/home.jsp). 

### Statistical analysis 

GraphPad Prism 6.0 software and R were performed for statistical analysis. Group comparisons were performed using the student's t-test or one-way ANOVA analysis. All data are presented as means + standard error (SEM). Differences with p-value < 0.05 were considered to be significant. Normality was analyzed with the Shapiro-Wilks test. Receiver operating characteristics (ROC) curves and area under the curve (AUC) were used to assess the prognostic properties of each lncRNA. MMSE score was compared between AD patients and controls using the Mann-Whitney test. The correlations between the variables were evaluated with the Spearman's correlation coefficient.

## Results

### Demographic and clinical features of the study subjects

Demographic and clinical features of the study population were summarized in Table 2(Tab. 2) (Supplementary data, Table 2). Fifty AD patients and fifty healthy controls were enrolled in the study. The *ApoE* genotype of the patients were determined by sequencing. The frequency of *ApoE4* allele was 26 % in the patient group and 4 patients were homozygote for this allele. The average age of AD and control groups were 76.65 ± 6.26 years, and 77.04 ± 6.03 years respectively. No significant differences were found between the AD patient and control groups in gender and mean age (p >0.05). However, both the MMSE (mini-mental state examination) score and education background in AD patients were significantly lower than those of the healthy controls (p < 0.05). 

### Plasma lncRNA profiling 

**Figure 1:**
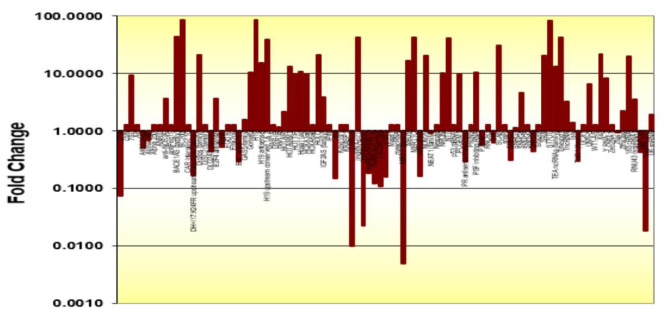
LncRNAs expression levels obtained by the human LncRNA Profiler qPCR Array Kit of which 42 were up-regulated but 23 were down-regulated in comparison with the healthy controls. Each bar represents an lncRNA.

The Human LncRNA Profiler qPCR Array Kit was used for profiling and screening of plasma lncRNAs. To this end, we first evaluated the expression levels of 90 different lncRNAs as well as five housekeeping genes including RNU43 snoRNA, 18S rRNA, U6B snRNA, GAPDH, Lamin A/C, in plasma samples of 10 AD and 10 healthy controls. The results showed that 4 of the internal control genes were not reproducible in all samples and only U6B snRNA was appropriate for usage as an internal normalizer gene. As shown in Figure 1(Fig. 1), out of the 90 lncRNAs screened, 42 were up-regulated, while 24 were down-regulated (Supplementary data, Figure 1). Then we selected 5 lncRNAs for further analysis based on the criteria; 1) its deregulation in brain tissues of the AD patients was already been reported, 2) we observed its repeated up- or down-regulation profile at least in 70 % of samples, 3) its fold change was ≥5. Most of the lncRNAs with *C_q_* value > 35 were excluded due to low abundance in plasma. Accordingly, lncRNAs BC200, NDM29, NEAT1, FAS-AS1 and GAS5-AS1 were selected as potential candidates for further investigation and validation in all samples.

### Analysis and validation of the candidate lncRNAs in all study subjects 

**Figure 2:**
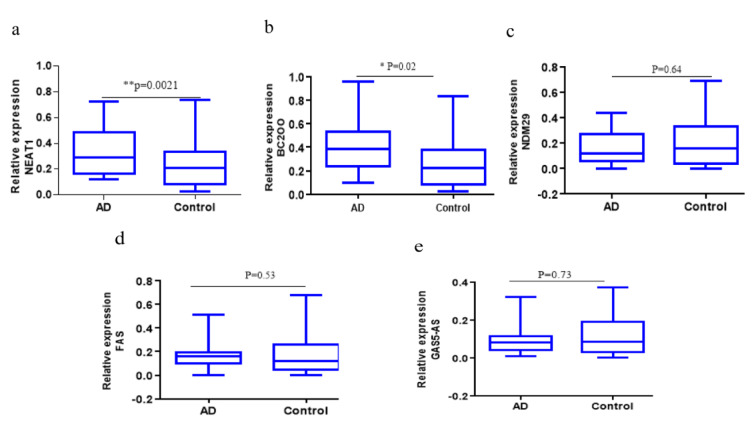
Plasma levels of the lncRNAs were quantified by qRT-PCR. The levels of a) NEAT1, b) BC200, c) NDM29, d) FAS-AS1 and e) GAS5-AS1 were compared in plasma samples of AD patients and healthy controls. A two-tailed unpaired t-test was performed.

**Figure 3:**
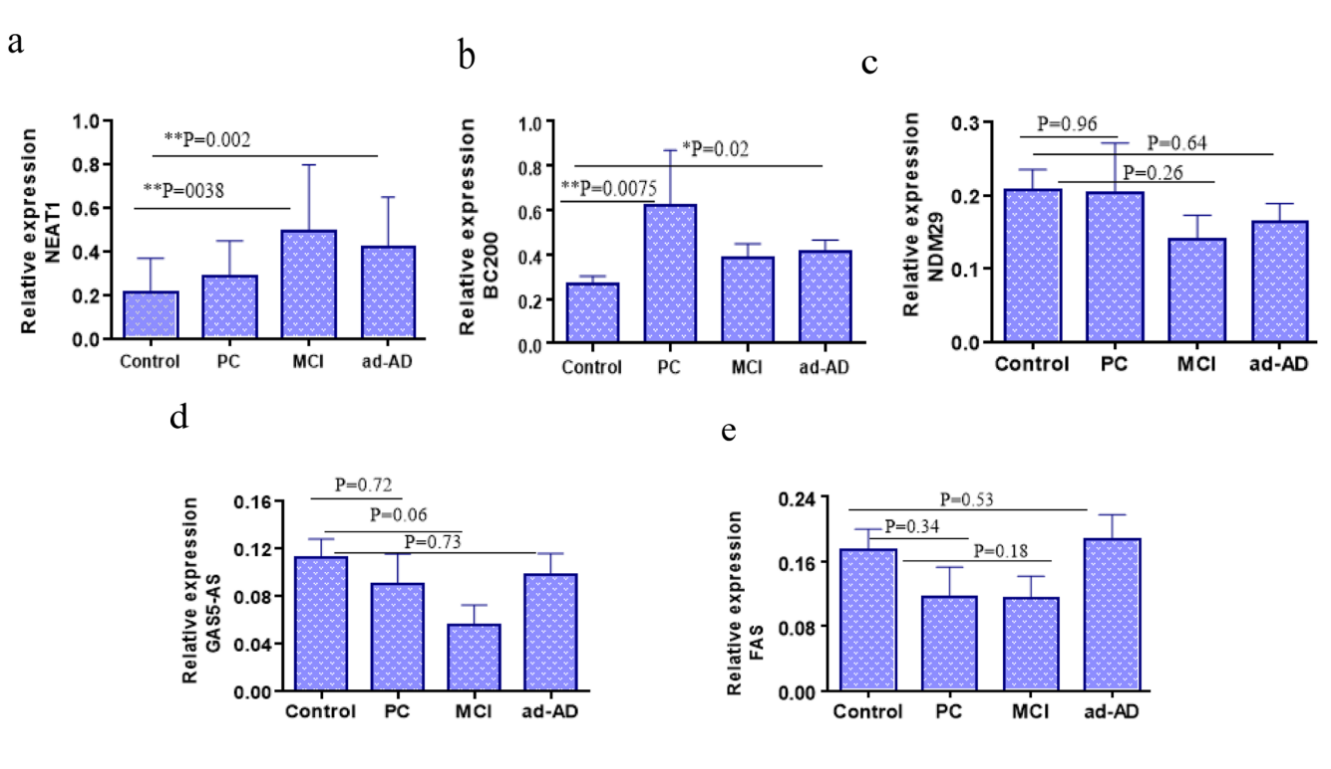
Comparison of the plasma levels of the studied lncRNAs between AD subgroups. The patients' group was divided into preclinical-AD (PC) (MMSE 24-26), MCI (MMSE 20-23) and advanced-AD (ad-AD) (MMSE<19) subgroups based on the corresponding MMSE scores of the subjects. Comparing the plasma levels of a) NEAT1 and b) BC200 between AD subgroups showed statistically significant differences. However, these comparisons for c) NDM29, d) GAS5-AS1 and e) FAS-AS1 did not show any significant differences. P values are listed above each chart.

To further validate reproducibility of the results obtained in the profiling phase of the study, plasma levels of the lncRNAs BC200, NDM29, NEAT1, FAS-AS1 and GAS5-AS1 in all AD and healthy controls were assessed by qRT-PCR and compared between two groups. As depicted in Figure 2(Fig. 2), the results revealed that the NEAT1 and BC200 levels in the plasma samples of the AD patients were significantly higher compared with the control group (p=0.0021 and p=0.02, respectively, Figure 2a and 2b(Fig. 2)). However, the plasma levels of lncRNAs NDM29, FAS-AS1 and GAS5-AS1 showed insignificant differences between two groups (p=0.64, p=0.53, p=0.73, respectively, Figure 2c-e(Fig. 2)). To determine whether the levels of these lncRNAs are correlated with the AD progression, we compared their levels between the control group and the PC (preclinical), MCI (Mild cognitive impairment) and ad-AD (advanced-AD) subgroups of the patient group (Figure 3(Fig. 3)). We observed significantly increased plasma levels of NEAT1 in the MCI and ad-AD subgroups compared to that of the control group (p=0.003 and p=0.002, respectively Figure 3a(Fig. 3)), but the differences between the AD subgroups were not statistically significant (Figure 3(Fig. 3)). Besides, plasma level of BC200 in the PC and ad-AD subgroup were significantly higher compared with the control group (p=0.007 and p=0.02, respectively), and there were not any significant differences between the subgroups (Figure 3b(Fig. 3)). Non-significant differences were found for the lncRNAs NDM29, FAS-AS1 and GAS5-AS1 in the comparisons between the PC, MCI, ad-AD and control groups (p>0.05) (Figure 3c-e(Fig. 3)).

### Effect of Apoε4 allele on the plasma levels of NEAT1 and BC200 

**Figure 4:**
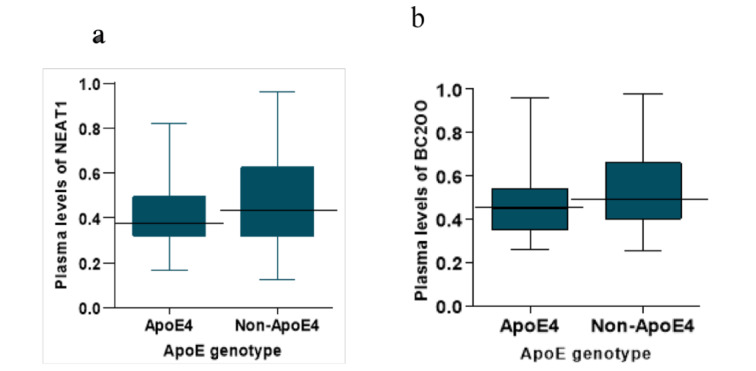
Effect of Apoε4 on the plasma levels of NEAT1 (a) and BC200 (b). The AD patients were scored as Apoε4 (with at least one Apoε4 allele) and non-Apoε4 (without Apoε4 allele), and the plasma levels of NEAT1 and BC200 were compared.

To understand whether Apoε4 allele might affect plasma levels of NEAT1 and BC200, genotypes of AD patients were determined by sequencing. Table 1(Tab. 1) shows the *ApoE* allele frequencies and genotypes of the AD patients. The Apoε4, Apoε3 and Apoε2 allele frequencies were 26 %, 72 % and 2 % respectively and 24 patients had at least one Apoε4 allele. Then we divided the patient group in two subgroups of Apoε4-positive and Apoε4-negative and compared the plasma levels of NEAT1 and BC200 between the two subgroups. These comparisons did not reveal any significant differences between the Apoε4-positive and Apoε4-negative subgroups (p=0.33 and p=0.30 respectively) (Figure 4 a, b(Fig. 4)).

### Biomarker potency of the plasma levels of lncRNAs NEAT1 and BC200 

**Figure 5:**
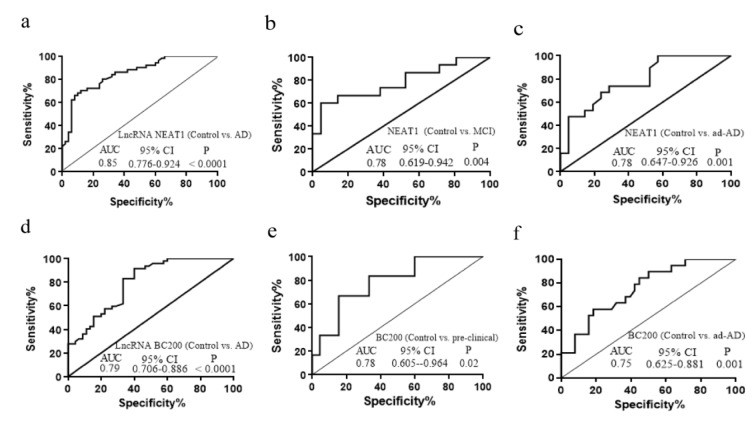
The ROC curve analysis for discriminative power of the plasma levels of lncRNAs. a) the NEAT1 level for AD versus control, b) MCI versus control and c) ad-AD versus control. ROC curve analysis on the BC200 level for d) AD versus control, e) pre-clinical versus control and f) ad-AD versus control. AUC = area under the ROC curve, ROC = receiver operating characteristic

To determine whether the plasma levels of NEAT1 and BC200 could play as blood-based biomarkers for AD, we conducted the receiver operating characteristic (ROC) curve analysis. As depicted in Figure 5(Fig. 5), ROC curve analysis on the NEAT1 level for AD versus control, leads to an AUC (area under the curves) of 0.85 (95 % CI: 0.776-0.924, p <0.0001) with 72 % sensitivity and 84 % specificity, indicating its good power on discriminating the AD patients from healthy controls (Figure 5a(Fig. 5)). We also assessed its power for discrimination between AD subgroups and control. The results showed that plasma level of NEAT1 could efficiently discriminate MCI (AUC=0.78, CI; 0.619-0.942, p=0.004) with 60 % sensitivity and 91 % specificity and ad-AD (AUC=0.78, CI; 0.647-0.926, p=0.001) with 73 % sensitivity and 71 % specificity from controls (Figure 5b, c(Fig. 5)).

ROC curve analysis on the BC200 level for AD versus control, leads to an AUC of 0.79 (95 % CI: 0.706 - 0.886, p<0.0001) with 60 % sensitivity and 91 % specificity, indicating that BC200 plasma level can discriminate AD patients from healthy controls (Figure 5d(Fig. 5)). We also evaluated its power for discrimination of AD subgroups and control. As Figure 5e(Fig. 5) shows, plasma level of BC200 could efficiently discriminate pre-clinical subjects from healthy controls (AUC=0.78, CI; 0.605-0.964, p=0.02) with 83 % sensitivity and 66 % specificity and ad-AD (AUC=0.75, CI; 0.625-0.881, p=0.001) patients with 84 % sensitivity and 55 % specificity (Figure 5f(Fig. 5)).

### Correlation of the plasma levels of NEAT1 and BC200 with age and MMSE 

**Figure 6:**
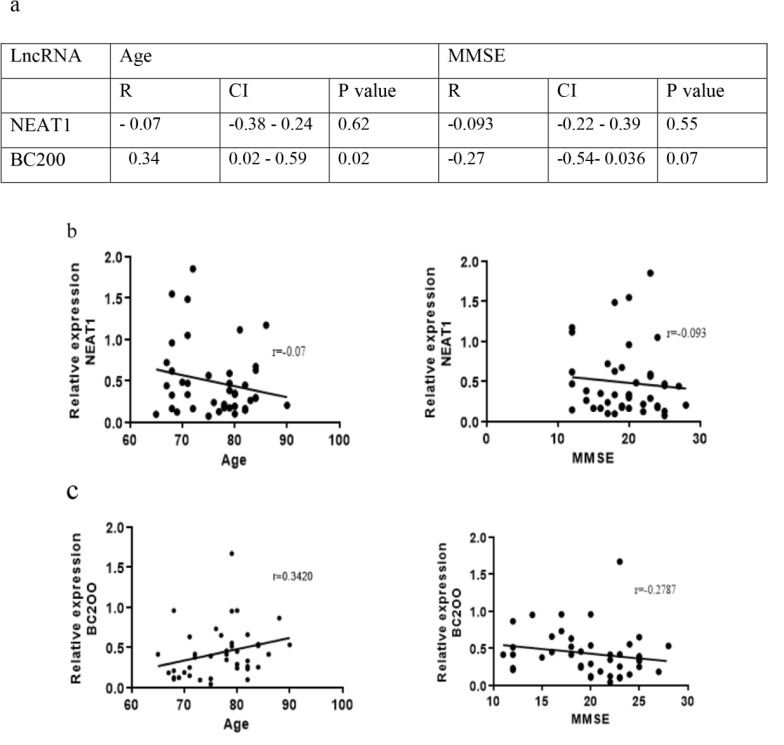
Correlation of the age and MMSE with plasma levels of NEAT1 and BC200 in AD patients. a) The correlation coefficient, CI and p values were outlined in the table. Correlations of the age or MMSE scores with b) NEAT1 and c) BC200 plasma levels. r; Spearman's correlation coefficient, CI; confidential interval

To understand whether the plasma levels of lncRNAs NEAT1 and BC200 correlate with the age or MMSE score of the AD patients, we used Spearman's rank correlation coefficient. The results were outlined in Figure 6a(Fig. 6). This analysis did not reveal significant correlations between NEAT1 level and age or MMSE score of the AD patients (Figure 6b(Fig. 6)). However, as depicted in Figure 6c(Fig. 6), the plasma level of BC200 showed a positive correlation with the age (r=0.34, CI; 0.02-0.59, p=0.02). Although we observed a negative correlation between BC200 level and the MMSE score of the AD patients, but this correlation was statistically rather non-significant (r=-0.27, CI; -0.54-0.036, p=0.07) (Figure 6c(Fig. 6)).

### Differentially expressed lncRNAs in AD

**Figure 7:**
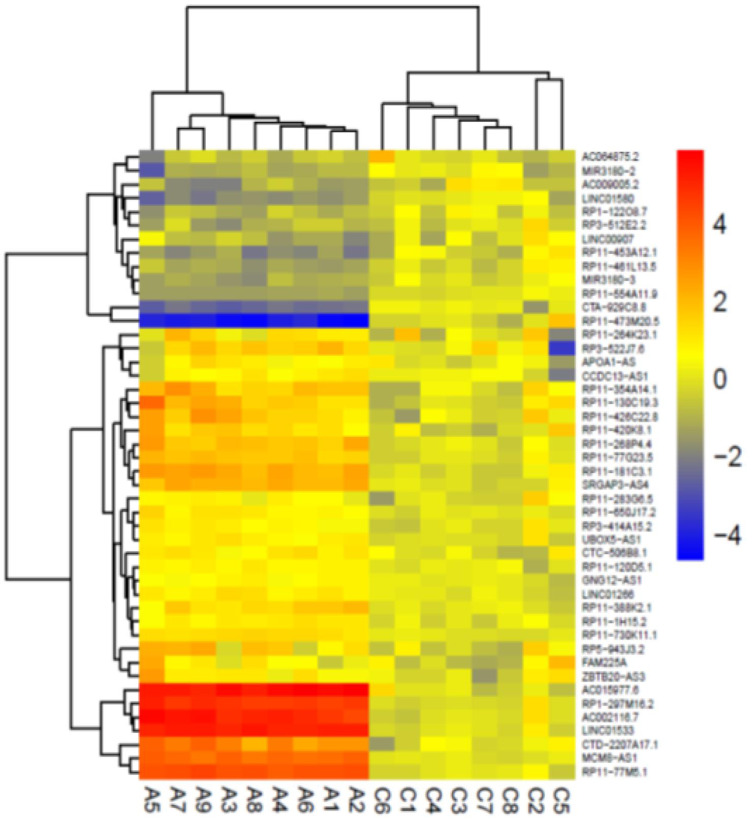
The heat map diagram of 46 differentially expressed lncRNAs in Alzheimer's disease samples obtained from reanalysis of the GSE53697 RNA-seq dataset. The color legend represents from low expression (blue) to high expression (Red). C; control, A; AD patient.

To further explore the differentially expressed lncRNAs in AD patients, we reanalyzed two datasets of RNA-seq. First, we reanalyzed the GSE136243 dataset including 164 plasma samples from AD patients. However, this analysis did not reveal any significant differences between the AD and controls for the studied lncRNAs. So, we focused on the GSE53697 dataset from human cerebral cortex tissues. We identified 33 up-regulated and 13 down-regulated lncRNAs by reanalyzing the GSE53697 dataset (p-value <0.05) (Supplementary data, Figure 7). Figure 7(Fig. 7) displays the heat map diagram of the differentially expressed lncRNAs between healthy people and AD patients.

### Gene set enrichment and pathway analysis 

Previous studies have confirmed that the lncRNAs are involved in the pathophysiological process of AD. Thus, the identification of the differentially expressed genes in AD patients might be helpful to realize the molecular mechanism of AD. To elucidate biological processes, cellular components, molecular functions and pathways which would be associated with aberrantly expressed lncRNAs in AD, enrichment analyses were performed for each lncRNA target. Results showed the gene ontology (GO) terms and pathways that among the most enriched of them were MAPK Signaling (false-discovery rate [FDR] = 0.00969), Toll-like Receptor Signaling (FDR = 0.0077), Visual Cycle (FDR = 0.00660), and Regulation of Innate Immunity (FDR = 0.0082) (see Supplementary data, Gene ontology terms and pathways). 

## Discussion

Emerging evidence revealed that lncRNAs play a pivotal role in neurodevelopment, brain function and aging (Roberts et al., 2014[30]). Increasing evidence also revealed that the dysregulation of lncRNA expression could be related to the AD pathogenesis (Khajehdehi et al., 2022[19]). Studies have reported some AD-associated lncRNAs, such as BACE1-AS, 51A, BDNF-AS and NAT-Rad18 that are aberrantly expressed in late-onset Alzheimer's disease (LOAD) compared with healthy controls (Abdolmaleki et al., 2021[1]; Cortini et al., 2019[6]). Expression profiling, using a comprehensive technical approach, also has characterized differentially expressed lncRNAs between AD samples and healthy controls in brain tissue of human (Annese et al., 2018[3]). Recently, with the advance of transcriptomics technology, circulating ncRNAs are increasingly used as alternative, novel and promising potential biomarkers for the early diagnosis of AD because of their noninvasive and easy accessibility (Galimberti et al., 2014[12]; Toden et al., 2020[37]). Several studies have focused on the circulating miRNAs and studied their biomarker potency for AD (Galimberti et al., 2014[12]; Zhao et al., 2019[45]). However, the expression pattern and diagnostic application of circulating lncRNAs in the plasma for neurodegenerative disorders such as AD have rarely been studied (Feng et al., 2018[10]; Fotuhi et al., 2019[11]). 

In this study, we first evaluated and compared the plasma levels of 90 lncRNAs between 10 AD patients and 10 healthy people, using a commercially available lncRNA array kit. Based on the results, we selected 5 lncRNAs (BC200, NDM29, NEAT1, FAS-AS1 and GAS5-AS1) as potential candidates for qPCR validation in the total cohort consisting of 50 AD and 50 healthy controls to measure the relative expression. The results showed that expression levels of NEAT1 and BC200 lncRNAs were significantly up-regulated in plasma of Alzheimer's patients compared to healthy volunteers. Moreover, ROC curve was plotted to evaluate the biomarker potential of NEAT1 and BC200 lncRNAs for Alzheimer's disease. The area under the curve with high specificity and sensitivity indicated that these lncRNAs can be considered as biomarkers for Alzheimer's disease. Besides, no correlation was found between the expression levels of the lncRNAs NDM29, FAS-AS1, GAS5-AS1, BC200 and the age or MMSE score of the patients. 

These findings are in agreement with results of animal model's research considering NEAT1 function involving in epigenetic regulation mechanisms in AD pathology (Asadi et al., 2021[4]). Huang et al. found that lncRNA NEAT1 was elevated during aging in the APP/PS1 mouse model (Huang et al., 2020[16]). Up-regulation of NEAT1 increased ubiquitination, led to inhibited autophagy signaling, gave rise to the amyloid accumulation and dysfunction of cognition (Huang et al., 2020[16]). A recent study showed lncRNA NEAT1 level was significantly higher in the temporal cortex and hippocampus of AD patients, indicating that NEAT1 was a biomarker for AD diagnosis (Spreafico et al., 2018[34]). RNA-seq analysis also showed a 3-fold up-regulation of NEAT1 in the temporal cortex and hippocampus of AD patients (Annese et al., 2018[3]). In this work, the expression level of lncRNA NEAT1 was measured and the results demonstrated that the plasma lncRNA NEAT1 level of AD patients was significantly higher than that of normal controls. These findings introduce NEAT1 as an attractive and promising molecular target that might be considered for AD intervention. 

The BC200 RNA acts as a translational regulator that modulates long-term synaptic plasticity (Li et al., 2018[21]). A study reported that BC200 levels in Aβ1-42 induced AD cell model are increased (Li et al., 2018[21]). They also showed that Knockdown of BC200 significantly suppressed BACE1 levels, increased cell viability and reduced cell apoptosis in AD cells, which can be reversed by BC200 overexpression (Li et al., 2018[21]). Additionally, Ahmadi and colleagues reported that lncRNA BC200 levels were up-regulated in AD brain (Ahmadi et al., 2020[2]). Moreover, our findings showed that the plasma lncRNA BC200 level of AD patients was significantly higher than that of healthy controls. Previous research suggested that altered relative BC200 levels up-regulated in the early stages of the disease (Ahmadi et al., 2020[2]; Asadi et al., 2021[4]). Similarly, we also found high expression of BC200 in early time point in the course of AD. Therefore, it may serve as a valuable biomarker in the early detection of Alzheimer's disease. Opposite to these results, Mus et al. showed that the expression of BC200 is significantly reduced in brain tissues from patients with AD compared with age-matched normal brains (Mus et al., 2007[28]). The reason for such discrepancy may be due to differences in brain regions chosen for the analysis and the severity of the disease (Asadi et al., 2021[4]). 

Furthermore, high-throughput experimental data obtained from GSE53697 dataset were reanalyzed. We found 46 differentially expressed genes, including 33 up-regulated and 13 down-regulated lncRNAs. GO terms were used to identify the molecular function represented in the gene profile by using the Database for Annotation, Visualization and Integrated Discovery (DAVID). Furthermore, we performed KEGG pathway analysis to determine the potential functions of these genes in biological processes and cellular components. Briefly, we introduced a set of lncRNAs that might be helpful for the study of AD pathophysiology.

In conclusion, the present study compared the plasma levels of 96 lncRNAs between AD and non-AD patients and found that the levels of NEAT1 and BC200 lncRNAs are increased in the plasma of AD patients. Plasma levels of both NEAT1 and BC200 efficiently discriminated the AD and healthy people highlighting their potential as new blood-based biomarkers for AD development. 

## Declaration

### Funding information 

This work was supported by the Cognitive Sciences and Technologies Council of Iran, grant number 6300.

### Declaration of competing interest 

The authors declare that they have no known competing financial interests or personal relationships that could have appeared to influence the work reported in this paper.

## Supplementary Material

Supplementary data

## Figures and Tables

**Table 1:**
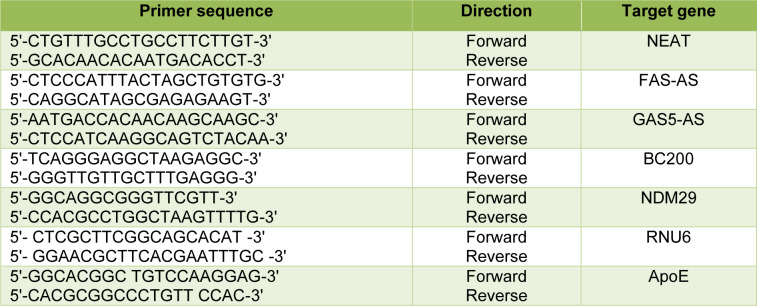
Sequences of the primers used in the study

**Table 2:**
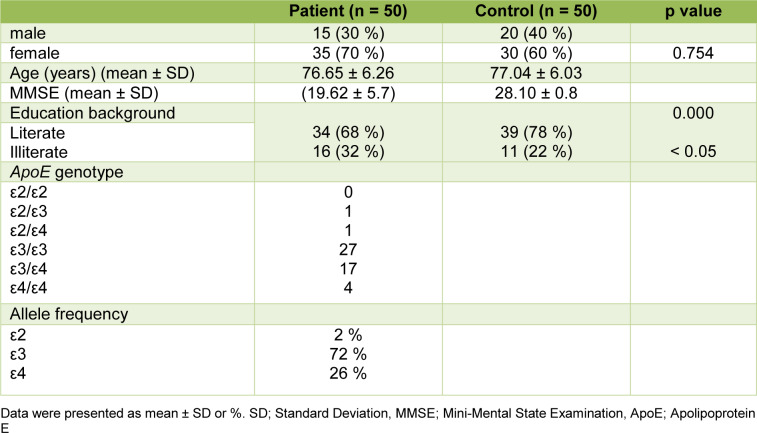
Demographic and clinical features of the study subjects
