# Supplementary material for: Plasma lncRNA profiling identified BC200 and NEAT1 lncRNAs as potential blood-based biomarkers for late-onset Alzheimer’s disease
Source: EXCLI J. 2022 May 9;21:772–85. doi: 10.17179/excli2022-4764 (PMC9360476; doi:10.17179/excli2022-4764)
Supplement: Supplementary data [file EXCLI-21-772-s-001.pdf]

EXCLI Journal 2022;21:772-785 – ISSN 1611-2156
Received: February 12, 2022, accepted: April 28, 2022, published: May 09, 2022

Supplementary data to:

Original article:

PLASMA LNCRNA PROFILING IDENTIFIED BC200 AND NEAT1 LNCRNAS AS POTENTIAL BLOOD-BASED BIOMARKERS FOR LATE-ONSET ALZHEIMER'S DISEASE

Majid Khodayi[1], Mohammad Khalaj-Kondori[1,*], Mohammad Ali Hoseinpour Feizi[1], Mortaza Jabarpour Bonyadi[1], Mahnaz Talebi[2]

1 Department of Animal Biology, Faculty of Natural Sciences, University of Tabriz, Tabriz, Iran
2 Neurosciences Research Center, Tabriz University of Medical Sciences, Tabriz, Iran

* **Corresponding author:** Mohammad Khalaj-Kondori, Department of Animal Biology, Faculty of Natural Sciences, University of Tabriz, Tabriz, Iran. Tel. +984133392674; E-mail: khalaj@tabrizu.ac.ir

https://dx.doi.org/10.17179/excli2022-4764

This is an Open Access article distributed under the terms of the Creative Commons Attribution License (http://creativecommons.org/licenses/by/4.0/).

Raw data to Table 2: Demographic data including age, gender, educational level, Mini-Mental State Examination score, and Apolipoprotein E genotype, were collected for all subjects. Fifty AD patients and fifty healthy controls were enrolled in the study.

Patient	Age	Gender	Education	MMSE	ApoE genotype	Control	Age	Gender	Education	MMSE
**1**	82	F	Literate	16	ε2/ε3	1	70	F	Illiterate	28
**2**	72	F	Literate	15	ε2/ε4	2	64	M	Literate	29
**3**	84	M	Literate	23	ε3/ε3	3	67	M	Literate	28
**4**	84	M	Literate	19	ε3/ε3	4	75	F	Literate	29
**5**	83	F	Illiterate	14	ε3/ε3	5	81	M	Literate	27
**6**	82	F	Literate	12	ε3/ε3	6	75	F	Illiterate	28
**7**	67	M	Literate	17	ε3/ε3	7	86	F	Literate	28
**8**	77	F	Literate	25	ε3/ε3	8	84	M	Illiterate	27
**9**	68	F	Literate	12	ε3/ε3	9	85	M	Literate	28
**10**	68	F	Illiterate	22	ε3/ε3	10	83	F	Illiterate	28
**11**	82	M	Literate	23	ε3/ε3	11	69	F	Literate	29
**12**	68	F	Literate	21	ε3/ε3	12	79	F	Literate	29
**13**	77	F	Literate	25	ε3/ε3	13	72	F	Literate	29
**14**	78	F	Illiterate	23	ε3/ε3	14	75	M	Literate	29
**15**	78	M	Literate	19	ε3/ε3	15	82	M	Literate	27
**16**	80	F	Illiterate	21	ε3/ε3	16	65	F	Literate	29
**17**	69	F	Literate	22	ε3/ε3	17	74	M	Literate	29
**18**	84	F	Illiterate	21	ε3/ε3	18	74	F	Literate	28
**19**	84	F	Illiterate	18	ε3/ε4	19	74	F	Literate	29
**20**	88	M	Literate	12	ε3/ε4	20	80	F	Illiterate	27
**21**	70	M	Illiterate	21	ε3/ε4	21	67	M	Literate	29
**22**	71	M	Literate	25	ε3/ε4	22	84	F	Literate	29
**23**	78	F	Illiterate	24	ε3/ε4	23	85	F	Illiterate	27
**24**	71	F	Literate	18	ε3/ε4	24	88	F	Literate	27
**25**	75	M	Literate	25	ε3/ε4	25	72	M	Literate	28
**26**	86	M	Illiterate	12	ε3/ε4	26	73	F	Literate	28
**27**	65	F	Literate	18	ε4/ε4	27	78	M	Literate	28
**28**	68	F	Illiterate	20	ε4/ε4	28	72	F	Literate	27
**29**	75	F	Literate	23	ε4/ε4	29	75	M	Literate	29
**30**	67	F	Literate	25	ε3/ε4	30	85	F	Illiterate	27
**31**	79	F	Literate	24	ε3/ε4	31	75	F	Literate	29
**32**	81	M	Literate	13	ε3/ε3	32	85	M	Literate	28
**33**	72	F	Literate	23	ε3/ε3	33	75	M	Literate	29
**34**	73	F	Literate	23	ε3/ε3	34	66	F	Illiterate	28
**35**	71	M	Literate	18	ε3/ε3	35	79	M	Literate	29
**36**	82	F	Illiterate	19	ε3/ε3	36	82	M	Literate	27
**37**	71	F	Literate	24	ε3/ε3	37	73	F	Literate	28
**38**	82	M	Illiterate	25	ε3/ε3	38	73	F	Literate	29
**39**	76	F	Literate	18	ε3/ε3	39	72	F	Literate	27
**40**	80	F	Illiterate	19	ε3/ε3	40	82	M	Illiterate	27
**41**	80	F	Literate	16	ε3/ε3	41	80	F	Literate	28
**42**	80	F	Literate	17	ε3/ε3	42	82	M	Illiterate	27
**43**	79	M	Illiterate	14	ε3/ε4	43	76	F	Literate	29
**44**	78	F	Literate	23	ε3/ε4	44	80	F	Illiterate	27
**45**	79	F	Literate	23	ε3/ε4	45	85	M	Literate	28
**46**	90	M	Illiterate	25	ε3/ε4	46	80	F	Literate	28
**47**	75	F	Illiterate	21	ε3/ε4	47	79	F	Literate	28
**48**	68	F	Literate	20	ε3/ε4	48	78	F	Literate	29
**49**	79	F	Literate	14	ε3/ε4	49	79	F	Literate	28
**50**		F	Literate	11	ε4/ε4	50	78	M	Literate	28

M= Male, F= Female, MMSE= Mini-Mental State Examination, ApoE= Apolipoprotein E

EXCLI Journal 2022;21:772-785 – ISSN 1611-2156
Received: February 12, 2022, accepted: April 28, 2022, published: May 09, 2022

Raw data to Figure 1: The fold changes of 90 different lncRNAs as well as five housekeeping genes including RNU43 snoRNA, 18S rRNA, U6B snRNA, GAPDH, Lamin A/C, in plasma samples of 10 AD and 10 healthy controls were obtained by the human LncRNA Profiler qPCR Array Kit.

Well	lncRNAs	Fold change
**A1**	21A	0.0757
**A2**	7SK	1.2634
**A3**	7SL	9.3655
**A4**	Air	1.2634
**A5**	AK023948	0.5203
**A6**	Alpha 280	0.6865
**A7**	Alpha 250	1.2634
**A8**	ANRIL	1.2634
**A9**	anti-NOS2A	3.6486
**A10**	antiPeg11	1.2634
**A11**	BACE1AS (family)	43.937
**A12**	BC200	84.294
**B1**	CAR Intergenic 10	1.2634
**B2**	DHFR upstream transcripts (family)	0.1716
**B3**	Dio3os (family)	20.783
**B4**	DISC2 (family)	1.2634
**B5**	DLG2AS (family)	0.4436
**B6**	E2F4 antisense	3.6234
**B7**	EgoA	0.5239
**B8**	EGO B	1.2634
**B9**	Emx2os	1.2634
**B10**	Evf1 and EVF2	0.2968
**B11**	GAS5-family	1.5447
**B12**	Gomafu	10.537
**C1**	H19	84.294
**C2**	H19 antisense	15.005
**C3**	H19 upstream conserved 1 & 2	38.515
**C4**	HAR1A	1.2634
**C5**	HAR1B	1.1626
**C6**	HOTAIR	2.1248
**C7**	HOTAIRM1	13.337
**C8**	HOTTIP	9.8311
**C9**	Hoxa11as	10.61
**C10**	HOXA3as	9.8995
**C11**	HOXA6as	1.2634
**C12**	HULC	21.22
**D1**	IGF2AS (family)	3.9105
**D2**	IPW	1.2634
**D3**	Jpx	0.1494
**D4**	Kcnq1ot1	1.2634
**D5**	KRASP1	1.2634
**D6**	L1PA16	0.01
**D7**	lincRNA-p21	42.44
**D8**	lincRNA-RoR	0.0225
**D9**	lincRNA-SFMBT2	0.1878
**D10**	lincRNA-VLDLR	0.1239
**D11**	LOC285194	0.1086
**D12**	LUST	0.1612

Raw data to Figure 1 (cont.): The fold changes of 90 different lncRNAs as well as five housekeeping genes including RNU43 snoRNA, 18S rRNA, U6B snRNA, GAPDH, Lamin A/C, in plasma samples of 10 AD and 10 healthy controls were obtained by the human LncRNA Profiler qPCR Array Kit.

Well	lncRNAs	fold change
**E1**	Malat1	1.2634
**E2**	mascRNA	1.2634
**E3**	MEG3 (family)	0.0049
**E4**	MEG9	16.534
**E5**	MER11C	42.147
**E6**	ncR-uPAR	0.1646
**E7**	NDM29	20.64
**E8**	NEAT1 (family)	0.9122
**E9**	Nespas	1.2811
**E10**	NRON	10.038
**E11**	NTT	41.567
**E12**	p53 mRNA	1.2634
**F1**	PCGEM1	9.8995
**F2**	PR antisense transcripts	0.2927
**F3**	PRINS	1.2634
**F4**	PSF inhibiting RNA	10.392
**F5**	PTENP1	0.5853
**F6**	RNCR3	1.2634
**F7**	SAF	0.623
**F8**	SCA8	30.64
**F9**	snaR	1.29
**F10**	SNHG1	0.3181
**F11**	SNHG3	1.1387
**F12**	SNHG4	4.6183
**G1**	SNHG5	1.2634
**G2**	SNHG6	0.4405
**G3**	Sox2ot	1.2634
**G4**	SRA	20.075
**G5**	ST7OT	83.133
**G6**	TEA ncRNAs (family)	13.337
**G7**	Tmevpg1	42.44
**G8**	TncRNA	3.2207
**G9**	Tsix	1.373
**G10**	TUG1 (family)	0.303
**G11**	UCA1	1.2634
**G12**	UM9-5	6.5312
**H1**	WT1-AS	1.2634
**H2**	Xist	21.368
**H3**	Y RNA-1	8.2669
**H4**	Zeb2NAT	1.2634
**H5**	Zfas1	0.9443
**H6**	Zfhx2as	2.1846
**H7**	18S rRNA	1.8592
**H8**	RNU43 (snoRNA)	2.1347
**H9**	GAPDH	0.6512
**H10**	LAMIN A/C	0.7173
**H11**	U6 snRNA	1.0127

Raw data to Figure 7: The following table lists log2 fold changes and *p values* of the 46 differentially expressed lncRNAs (33 up-regulated and 13 down-regulated). If the log2 fold change value is higher than 1, that gene has increased in the Alzheimer's sample compared to the normal sample, and if the log2 fold change value is less than 0, that gene has decreased in the Alzheimer's sample compared to the normal sample. p-value < 0.05 was considered statistically significant.

LncRNA	log2foldchange	p-value
AC015977.6	5.021876	0.040735
AC002116.7	4.302559	0.040735
LINC01533	4.079406	0.01525
RP1-297M16.2	3.924566	0.040735
RP11-77M5.1	3.415254	0.00291
CTD-2207A17.1	2.818729	0.040735
MCM8-AS1	2.812686	0.02881
RP11-181C3.1	2.261071	0.01851
SRGAP3-AS4	2.070602	0.049145
RP11-130C19.3	1.942549	0.001645
RP11-268P4.4	1.902838	0.015735
RP11-426C22.8	1.818981	0.036895
RP11-354A14.1	1.7962	0.039575
RP11-77G23.5	1.669089	0.036895
RP11-388K2.1	1.363121	0.039925
RP11-420K8.1	1.318804	0.029875
RP3-522J7.6	1.30376	0.029875
RP11-650J17.2	1.194805	0.03949
RP11-1H15.2	1.18612	0.01234
RP5-943J3.2	1.179981	0.040505
RP11-730K11.1	1.17893	0.00288
RP11-264K23.1	1.142734	0.00288
UBOX5-AS1	1.125041	0.03949
RP11-283G6.5	1.08542	0.03949
CCDC13-AS1	1.065485	0.02756
RP11-120D5.1	1.056605	0.004935
APOA1-AS	1.042099	0.01851
FAM225A	1.026811	0.03949
CTC-506B8.1	1.019807	0.001645
RP3-414A15.2	1.016449	0.02756
LINC01266	1.013907	0.01234
GNG12-AS1	1.011789	0.01851
ZBTB20-AS3	1.009658	0.03949
LINC00907	-1.00639	0.001645
RP1-122O8.7	-1.05226	0.03949
AC064875.2	-1.07047	0.01234
RP3-512E2.2	-1.08153	0.03949
RP11-461L13.5	-1.21311	0.03949
MIR3180-3	-1.25054	0.007815
MIR3180-2	-1.26425	0.007815
AC009005.2	-1.30897	0.01851
RP11-554A11.9	-1.30987	0.03949
RP11-453A12.1	-1.78919	0.005905
LINC01580	-1.94511	0.005495
CTA-929C8.8	-2.57173	0.03034
RP11-473M20.5	-4.10312	0.004195

Abbreviations: SRGAP3-AS4= SRGAP3 antisense RNA 4; MIR3180-2= MicroRNA 3180-2; FAM225A= Family with Sequence Similarity 225 Member A; CCDC13-AS1= CCDC13 Antisense RNA 1; LINC00907= Long Intergenic Non-Protein Coding RNA 907; MCM8-AS1= MCM8 Antisense RNA 1; UBOX5-AS1= UBOX5 Antisense RNA 1; APOA1-AS= APOA1 Antisense RNA; ZBTB20-AS3= ZBTB20 Antisense RNA 3

EXCLI Journal 2022;21:772-785 – ISSN 1611-2156
Received: February 12, 2022, accepted: April 28, 2022, published: May 09, 2022

Raw data to Gene ontology terms and pathways: Pathway analysis of the differentially expressed lncRNAs obtained from GSE53697 RNA-seq dataset revealed potential involved pathways. LncRNAs and the most enriched pathways were listed here in the Table.

lncRNA	pathway
SRGAP3-AS4	The Visual Cycle
SRGAP3-AS4	Retinoate Biosynthesis I
CTD-2207A17.1	Chondroitin Sulfate Degradation
CTD-2207A17.1	Dermatan Sulfate Degradation
MIR3180-2	p38 MAPK Signaling
MIR3180-3	p38 MAPK Signaling
FAM225A	The Visual Cycle
CCDC13-AS1	The Visual Cycle
RP11-130C19.3	Chondroitin Sulfate Degradation
RP11-130C19.3	Dermatan Sulfate Degradation
RP11-130C19.3	The Visual Cycle
LINC00907	Role of Oct4 in Mammalian Embryonic Stem Cell Pluripotency
RP11-77G23.5	Chondroitin Sulfate Degradation
RP11-77G23.5	Dermatan Sulfate Degradation
RP11-77G23.5	The Visual Cycle
MCM8-AS1	iNOS Signaling
MCM8-AS1	Toll-like Receptor Signaling
RP1-122O8.7	iNOS Signaling
RP1-122O8.7	Toll-like Receptor Signaling
RP11-120D5.1	Cell Cycle Control of Chromosomal Replication
CTD-2207A17.1	Glioma Invasiveness Signaling
RP11-130C19.3	Retinoate Biosynthesis I
RP5-943J3.2	MIF-mediated Glucocorticoid Regulation
RP5-943J3.2	MIF Regulation of Innate Immunity
RP5-943J3.2	Phospholipases
RP5-943J3.2	Eicosanoid Signaling
RP5-943J3.2	Role of MAPK Signaling in the Pathogenesis of Influenza
RP5-943J3.2	VEGF Family Ligand-Receptor Interactions
MCM8-AS1	IL-1 Signaling
RP1-122O8.7	IL-1 Signaling
RP5-943J3.2	Antioxidant Action of Vitamin C
RP3-512E2.2	Leukocyte Extravasation Signaling
RP3-512E2.2	Chondroitin Sulfate Degradation
RP3-512E2.2	Dermatan Sulfate Degradation
RP11-77M5.1	MIF-mediated Glucocorticoid Regulation
RP11-77M5.1	MIF Regulation of Innate Immunity
RP11-77M5.1	Phospholipases
RP11-77M5.1	Eicosanoid Signaling
RP11-77M5.1	Role of MAPK Signaling in the Pathogenesis of Influenza

Abbreviations: SRGAP3-AS4= SRGAP3 antisense RNA 4; MIR3180-2= MicroRNA 3180-2; FAM225A= Family with Sequence Similarity 225 Member A; CCDC13-AS1= CCDC13 Antisense RNA 1; LINC00907= Long Intergenic Non-Protein Coding RNA 907; MCM8-AS1= MCM8 Antisense RNA 1; p38 MAPK= p38 mitogen-activated protein kinases; Oct4= Octamer-binding transcription factor 4; iNOS Signaling= inducible nitric oxide synthase Signaling; MIF= Macrophage migration inhibitory factor; VEGF= Vascular endothelial growth factor; IL-1= Interleukin-1; UBOX5-AS1= UBOX5 Antisense RNA 1; APOA1-AS= APOA1 Antisense RNA; ZBTB20-AS3= ZBTB20 Antisense RNA 3
